# Brief overview of meningitis in the Pacific Islands, and implications for public health, clinical, and rehabilitation services: A call to action

**DOI:** 10.7189/jogh.11.03006

**Published:** 2021-01-16

**Authors:** Annette Kaspar, Sione Pifeleti

**Affiliations:** 1 ENT Department, Tupua Tamasese Meaole Hospital, Ministry of Health, Apia, Samoa; 2 Hearing Research Unit for Children, School of Health and Rehabilitation Sciences, University of Queensland, Brisbane, Australia

Meningitis is a life-threatening inflammation of the membranes covering the brain and spinal cord [1]. It is usually caused by bacterial and viral pathogens, and is more commonly found among children experiencing malnutrition, overcrowded households, and low levels of immunization [1]. Patients who survive meningitis infection are at risk of developing permanent disabilities, including physical, hearing, and visual impairments [2]. In Low and Middle Income Countries, improvements in public health, and the expansion of clinical and rehabilitation services for people with disabilities should reduce the burden of meningitis disease and its associated impairments. While the research appropriately focuses on improving immunization programs to prevent meningitis, there is little attention given to people currently living with the consequences of the infection. The World Health Organization highlighted the importance of improving diagnostic and rehabilitation services by including ‘Support and Care for people affected by meningitis’ as the fourth of five pillars in their ‘Defeating Meningitis Roadmap’ [3].

There is very little in the research literature on meningitis in the Pacific Islands. Encouragingly, the most recent report from the Global Burden of Disease Study found that the incidence, deaths, and Disability-Adjusted Life Years (DALYs) for meningitis in the Pacific Islands are in decline [1]. This may be attributed to improvements in routine childhood immunization programmes under the Millennium Development Goal Project (2000-2015), which targeted the meningitis bacterial pathogens *Haemophilus influenzae* Type b, *Streptococcus pneumoniae*, and *Neisseria meningitidis* (**Table 1**). However, meningitis is still a cause of death and disability in regions where immunization coverage is below optimal levels (**Table 1**).

**Table 1:** Availability of immunizations against meningitis, and coverage rate for immunizations including the *Haemophilus influenzae* type B vaccine in the Pacific Islands [4]

	Meningitis pathogen			Hib vaccine delivery and coverage rate		
**Country**	***Neisseria meningitidis***	***Streptococcus pneumoniae***	***Haemophilus influenzae* type B**	**Hib vaccine**	**1^st^ dose coverage rate (%)**	**3^rd^ dose coverage rate (%)**
Cook Islands			Yes	DTaPHibHepB	99	99
Fiji		Yes	Yes	DTwPHibHepB	99	99
Kiribati		Yes	Yes	DTwPHibHepB	98	95
Marshall Islands	Yes	Yes	Yes	DTaPHibIPV	97	81
Micronesia		Yes	Yes	Hib	-	59
Nauru			Yes	DTwPHibHepB	99	90
Niue		Yes	Yes	Hib	-	99
Palau		Yes	Yes	Hib	-	92
Papua New Guinea		Yes	Yes	DTwPHibHepB	67	61
Samoa			Yes	DTwPHibHepB	56	34
Solomon Islands		Yes	Yes	DTwPHibHepB	86	85
Tonga			Yes	DTwPHibHepB	86	81
Tuvalu			Yes	DTwPHibHepB	99	89
Vanuatu			Yes	DTwPHibHepB	93	85

DTaPHibHepB – diphtheria and tetanus and pertussis and *Haemophilus influenzae* type B and hepatitis B vaccine, DTwPHibHepB – pentavalent diphtheria and tetanus toxoid with pertussis, *Haemophilus influenzae* type B and hepatitis B vaccine vaccine, DTaPHibIPV – diphtheria and tetanus toxoid with acellular pertussis, *Haemophilus influenzae* type B and inactivated polio (IPV) vaccine

A recent paper published in this journal called attention to the role of otorhinolaryngology and ophthalmology services in the aftermath of the measles epidemic in the Pacific Islands [5]. The aim of this brief perspective is to similarly highlight meningitis and the implications for public health and clinical services in the Pacific Islands. In parallel to improved immunization coverage to reduce the rate of meningitis among children, the development of essential clinical and rehabilitations services for meningitis survivors is advocated.

**Figure:**
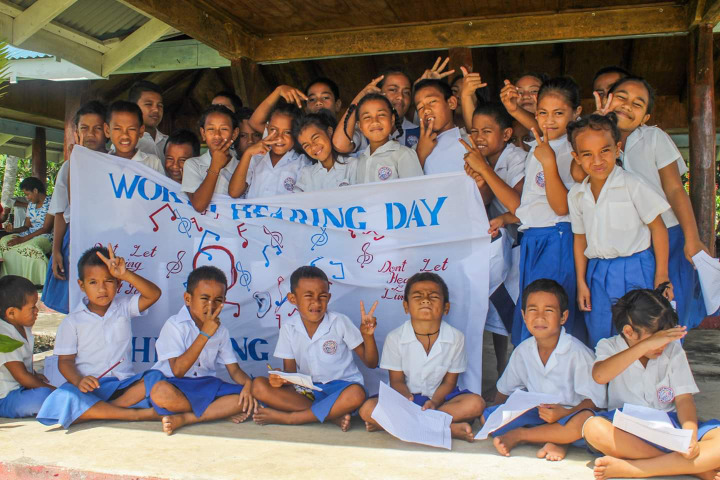
Photo: From the author’s own collection, used with permission.

## OVERVIEW OF THE LITERATURE ON MENINGITIS IN THE PACIFIC ISLANDS

There is very little in the research literature on the epidemiology of meningitis in the Pacific Islands. A recent hospital-based study from Fiji found that *Streptococcus pneumoniae* (55%) was the most common bacterial pathogen for meningitis among children in the 0-5 age group, followed by *Neisseria meningitidis* (15%) and *Haemophilus influenzae* type b (10%) [6]. The study reported a case fatality rate of 36% for meningitis caused by *Streptococcus pneumoniae*. There was an outbreak of enterovirus at the time of the Fijian study, and this viral pathogen was reported as the most common cause of meningitis during the data collection time frame.

Another hospital-based study from Papua New Guinea investigated causal pathogens for suspected cases of meningitis among 1884 children between 1996 and 2005 [7]. Identifiable pathogens were isolated for 375 children only (19.9%), and revealed *Streptococcus pneumoniae* (n = 180) and *Haemophilus influenzae* (n = 153) as the leading causal microbial agents. This pattern, and their serotypes described in the study, may have altered since the introduction of the current immunization schedules.

A recent review of meningococcal disease in the Asia-Pacific region is available, however, the Pacific Islands were not represented in this study [8]. Information on the prevalence of *Neisseria meningitidis* would be beneficial for evidence-based decision-making regarding the introduction of immunization against this pathogen into routine immunization schedules in the Pacific Islands.

## OVERVIEW OF THE LITERATURE ON THE NEUROLOGICAL SEQUELAE OF MENINGITIS IN THE PACIFIC ISLANDS

A review of the literature found five papers addressing the leading neurological sequelae of meningitis among survivors in the Pacific Islands. There were four papers describing hearing impairment, with two of the papers published in the early 1990s [9,10], and the more recent publications were limited by sample size and lack of formal audiometry testing [6,11]. The early paper from Vanuatu reported a permanent hearing impairment rate of 32.3% among meningitis survivors, and it continues to be the most cited paper on meningitis in the Pacific Islands in the audiology literature. A common theme throughout the four papers retrieved, however, is that routine ear and hearing assessments should be performed for all meningitis survivors.

The fifth paper addressed physical disability and associated mobility challenges among meningitis survivors in Papua New Guinea [12]. The authors strongly advocated for improved clinical and rehabilitation services for meningitis survivors with physical and mobility difficulties, especially among people with disabilities living in rural and remote parts of the country. The authors also described physiotherapy as a young profession in Papua New Guinea, and called for greater education of Community-Based Rehabilitation Workers to meet the needs of the population majority living outside urban settings.

Visual impairment is another well-known neurological sequelae of bacterial meningitis. The present review did not find any papers describing visual disability among meningitis survivors in the Pacific Islands.

## IMPLICATIONS FOR PUBLIC HEALTH, CLINICAL AND REHABILITATION SERVICES IN THE PACIFIC ISLANDS

The table above indicates that there is an opportunity to strengthen routine childhood immunizations against the leading causal bacterial pathogens of meningitis in the Pacific Islands. Ideally, immunization should be available in all Pacific Island countries against the main bacterial pathogens of meningitis fatalities (*Neisseria meningitidis*) and meningitis-related disabilities (*Streptococcus pneumoniae*). At this time, all Pacific Islands include the *Haemophilus influenzae* vaccine in their immunization schedules, eight out of fourteen countries include the *Streptococcus pneumoniae* vaccine, and only the Marshall Islands offer the *Neisseria meningitidis* vaccine.

The barriers preventing optimal immunization coverage rates should also be addressed. The table indicates that the most recent coverage rate data was lowest for Samoa, and this is most likely related to the urgent recall of vaccines and necessary suspension of the National Immunization Program. The second lowest coverage rate is for Papua New Guinea, the largest archipelago of the Pacific Island countries, and therefore the nation with the greatest challenge of accessing all rural and remote areas for optimal immunization program delivery. This is an opportunity for cross-sector collaboration under the Sustainable Development Goals, where improved transport and infrastructure will also benefit health outcomes.

As advocated for the case of measles in the Pacific Islands, health promotion activities regarding meningitis in this region should highlight that routine childhood immunizations not only save lives, but also prevent the risk of life-long disabilities. The neurological sequelae of meningitis are well-known from the global literature. The Sustainable Development Goal Agenda offers an excellent platform to establish or strengthen clinical and rehabilitation services for meningitis survivors living with physical, hearing, and/or visual impairments. Among young children in particular, routine ear/hearing and eye/vision assessment is recommended because mild or moderate degrees of impairment may often be overlooked. Where specialized health professions are scarce or non-existent, the World Health Organization promotes Community-Based Rehabilitationists for the care of children and adults with disabilities.

## CONCLUSION

Although there is little in the research literature regarding meningitis and associated sequelae in the Pacific Islands, this should not deter efforts to strengthen current childhood immunization schedules, improve coverage rates, and include the leading causal meningitis pathogens of death and disability in the programme. Routine ear/hearing and eye/vision assessments for all meningitis survivors is also vital to ensure early identification of any sensory impairment, and facilitate early intervention options as required. Improved rehabilitation services for people with physical disabilities is also essential. As clinical and rehabilitation services are strengthened in urban settings, a regular outreach programme to rural/remote settings is strongly advocated.
